# Assessing the Nutrient Adequacy in China’s Food Supply from 1965 to 2018

**DOI:** 10.3390/nu13082734

**Published:** 2021-08-09

**Authors:** Ao Liu, Aixi Han, Li Chai

**Affiliations:** 1 International College Beijing, China Agricultural University, Beijing 100083, China; 2018314060506@cau.edu.cn (A.L.); 2017314060118@cau.edu.cn (A.H.); 2 College of Economics and Management, China Agricultural University, Beijing 100083, China

**Keywords:** nutrition, nutrient, public nutrition, food, food supply, China

## Abstract

Nutritional intake has important impacts on human health. A sufficient supply of nutrients is required to ensure high-level nutrition in a population. Assessment of nutrient supply adequacy can help to develop evidence-based policies and thereby promote public health. This study estimates the supply adequacy of nutrients in China’s food system from 1965 to 2018 at the national level, aiming to reveal whether the supply of nutrients meets the demand. The results show that the nutrient supply in China’s food system has experienced a sharp increase in the past five decades, and the deficiency in nutrient supply has been greatly mitigated. Although most nutrients such as potassium are already sufficiently supplied in China’s current food system, some nutrients, especially calcium and zinc, still need a further enlarged supply to improve the nutrition condition of the Chinese population. Besides encouraging a healthy diet, supply-side regulation, e.g., fortification and enrichment, is also needed to improve nutrient availability. This study helps people better understand the development and current situation of nutrient adequacy in China’s food supply, thereby providing information and implications for policymakers.

## 1. Introduction

China has experienced a dramatic diet transition over the past half a century. The per capita caloric supply of Chinese people increased from 1782 kcal/day in 1965 to 3200 kcal/day in 2018 [1], leading to a significant decreasing risk of hunger. The malnutrition prevalence in China has decreased from 24% in the early 1990s to less than 10% in recent years [2] thanks to the upgrading diet pattern, e.g., more intake of high-protein foods. According to FAO data, the per capita intake of meats has increased by almost 11 times over the past 50 years [1].

However, Chinese people are still facing nutritional imbalance issue as a consequence of unhealthy diet pattern, e.g., over intake of processed grains and red meats [3] but inadequate intake of coarse grains and dairy products [4] (Chang et al., 2018). As one of four major risk factors of non-communicable diseases, diet pattern greatly affects public health [5,6,7,8]. Unhealthy diet pattern has posed a great threat to China’s public health, e.g., osteoporosis [9,10], especially in elders, diabetes, obesity, and metabolic syndrome [11]. Thus, the diet-related health assessment is imperative for policymakers to identify the deficient or excessive intakes and design the related intervention policies. Although many previous studies have investigated, assessed, and analyzed the diet quality or nutrient intake, these studies are only for a specific population group, e.g., pregnant women [12], urban households [13], children and adolescents [14,15]. Some studies have examined the nutritional level and dietary quality based on China Health and Nutrition Survey (CHNS) [16,17,18]. However, CHNS only covers less than a half of the regions and therefore cannot represent for the national average [19]. These previous studies are helpful for policymakers to take pertinent interventions for the targeted people, but they are unable to show policymakers a macro picture in a national perspective and during a long period. 

Han et al. [20] evaluated the health score of Chinese diet based on dietary guidelines at the national level from 1961 to 2017. The authors found that, for Chinese diet, despite the severe deficiency has been significantly mitigated over the past 57 years, it is currently still lacking those high-quality and premium foods, such as dairy products. Tian et al. [21] examined the healthiness status of Chinese adults’ diet based on the Chinese Food Pagoda 2016; they also found that an imbalance exists in the current Chinese diet, i.e., over-consumption of cereals but under-consumption of vegetables and poultry. However, the aforementioned studies examined diet quality by investigating consumption of various food groups without also considering nutrient intakes. Understanding both the nutrient and food supplies of a country is informative to policymakers to improve public health. For instance, policymakers can enlarge the supply of those deficient nutrients by not only producing more nutrient-rich foods but also by fortifying the specific nutrient or providing more the specific nutrient supplementary. 

In order to advise China’s government how to further improve the nutritional level of its residents, this study investigates China’s nutrient supply adequacy in food supply based on the Food Balance Sheet of FAO and the GENuS database constructed by Smith et al. [22]. In this study, the total caloric intake is investigated to indicate whether there is enough energy available for Chinese people in their food supply; fifteen common nutrients are examined to reveal whether Chinese people are nutrition secure. The studied period is from 1965 to 2018, during which China’s food system has experienced a great transition [20,23]. This study aims to provide policymakers information and implications to further improve the nutritional level of Chinese people.

## 2. Materials and Methods

### 2.1. Nutrient Supply

The food supply data in this study were obtained from FAO food balance sheets (FBS). FAO food balance sheets (FBS) reported data on production, trade, and utilization of 98 food commodities for 175 current countries [1]. Instead of estimating consumption, FAO food balance sheets (FBS) estimates rough national availability of foods and selected nutrients (including energy, fat and protein). Its main advantage lies in providing global food supply coverage at the national level. After estimating the edible food supply of each country, the GENuS model of Harvard University matched the nutrition density in the food composition table to determine the per capita nutrient supply. This study covered 16 indicators, i.e., total calories and 15 specific nutrients, including protein, vitamin C, vitamin A, folate, calcium, iron, zinc, potassium, copper, phosphorus, thiamin, riboflavin, niacin, vitamin B5, and magnesium. These nutrients are of particular concern to China. Combining these two databases, we can get the food supply of nutrients of Chinese residents as follows:

(1) $$\begin{array}{c} NS_{i,n}=Food\;supply_{i}\times Nutrient_{i,\;n} \end{array}$$

where i represents different categories of food; n represents nutrients; $NS_{i,n}$ represents the nutrient supply of ***i***th food’s ***n***th nutrient; $Food\;supply_{i}$ represents the food supply data of ***i***th food from FAO-FBS; $Nutrient_{i,\;n}$ represents the content of ***n***th nutrients in ***i***th food from GENuS database.

The total nutrient supply from food system of Chinese residents can be obtained by multiplying the per capita nutrient supply data from different kinds of food (Equation (1)) by the total population. The equation is as follows:

(2) $$NS_{n}=TP\overset{19}{\underset{i=1}{\sum }}NS_{i,n}$$

where $NS_{n}$ represents the whole nutrient supply of ***i***th food’s ***n***th nutrient in China; TP represents the total population of China. 

### 2.2. Nutrient Demand

The nutrient demand differs a lot among the different population groups, especially among ages and genders. The national nutrient demand is calculated based on the nutrient demand of each specific group (e.g., children) and the population of this group. Due to data availability, this study only considers the population groups by age and gender but does not consider the special groups, such as pregnant women and patients. 

We obtained the per capita daily demand of nutrients from China Food Composition Tables and got the population structure data (by gender and age) of China during 1965 to 2018 from the World Population Prospects [24]. By summing up the nutrient demands of different genders and ages, we calculate the annual per capita nutrient demand of Chinese residents from 1965 to 2018 as follows:

(3) $$ND_{n}=DRI_{a,n}\times P_{a}\times 365$$

where a refers to the ath age group (including different gender); $ND_{n}$ indicates the annual demand for ***n***th nutrient per capita; $DRI_{a,n}$ refers to the ***a***th age group’s daily reference intake for ***n***th nutrient from China’s food composition table. The reference level of DRI in this study indicates a level that is expected to meet the need of about half the healthy population of that life-stage group; $P_{a}$ is the population of ***a***th age group (including different gender).

### 2.3. Supply Adequacy

Nutrient supply adequacy is defined as the ratio of supply (i.e., available nutrients in food supply) to demand (i.e., recommended intake amount of nutrients). It can be calculated as the following equation:

(4) $$r_{n}=\frac{NS_{n}}{ND_{n}}$$

where $r_{n}$ is the supply adequacy of the ***n***th nutrient.

Dietary evaluation is usually based on an intake target for a certain population. Population nutrient intake goals refer to the average intake of healthy people, and the health standard is marked by the low prevalence of diet related diseases. If the existing population average is below this range, health problems may occur. Therefore, there is rarely a single “optimal value” for nutrient intake target, which leads to the comparison of nutrient supply adequacy around one.

Because of uneven distribution, there will always be a part of population intake insufficient and another part of people intake excessive, even if the supply and demand reach a balance situation (the ratio equals one). Therefore, a ratio of one (in Table 1) only means that supply and demand are just in balance. Considering the problem of uneven distribution, there is no upper limit to the ratio of supply and demand of nutrients, that is, more is better.

The daily nutrient intake per capita of Chinese residents can be generated by accurately converting the food intake into nutrients to evaluate Chinese residents’ nutritional status. Generally, under the condition of average distribution, a ratio of less than one means the nutrient demand is greater than the supply, which means that there is an inadequate intake of nutrients. A ratio equal to one indicates that nutrient supply and demand balance, although uneven distribution may still exist. A ratio that exceeds one might result in surplus consumption because the supply of nutrients is greater than the demand. The larger the proportion is, the larger the consumer surplus is and therefore, the supply of this nutrient is only sufficient for a group of people.

The relevant explanations of the ratio of nutrient supply to demand are as follows:

## 3. Results

### 3.1. The Food Supply Structure of Chinese Residents

**Figure 1:**
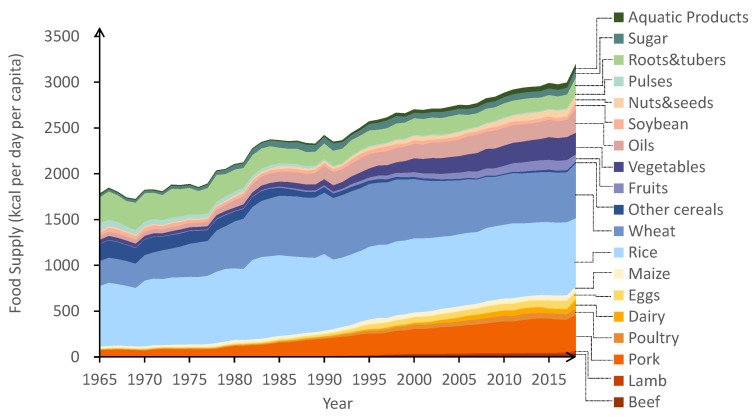
The supply of different food categories of Chinese residents from 1965 to 2018.

Figure 1 demonstrates the change in the average daily food supply of Chinese residents from 1965 to 2018. The consumption of all food categories doubled from 1782 kcal per day in 1965 to 3200 kcal per day in 2018. Among them, staple foods (including maize, rice, wheat, and other cereals) accounted for the most significant proportion of the total food supply, with an average of 60% in the total 54 years. The proportion of staple foods increased considerably before dropping. From 1965 to 1985, it increased from 64% to 79%, and then gradually decreased, reaching 44% in 2018. The consumption of soybeans, nuts and tubes also experienced a slow decline over these years. With improved living conditions, their proportion among the whole food supply decreased from 24% in 1965 to 8.5% in 2006. However, the consumption of soybeans, roots and tubes increased to 11% in 2018.

Meat including beef, lamb, pork and poultry also witnessed an almost fourfold increase, from 4% in 1965 to 17% in 2018. Similarly, the share of aquatic products was growing year on year. The aquatic products intake only accounted for 0.6% of 1965 before it already hit 1.9% till 2017. Vegetables and fruits increased from 2.7% to 12% among these five decades.

In contrast, the availability of oil and sugar went through a different curve. Between 1965 to 2017, the oil rose by 4.8% from 2.4% before falling. Similarly, sugar consumption almost doubled from 1.5% in 1965 to 3% in 1987. However, it decreased to 2.4% in 1999 and fluctuated around this value.

Overall, the structure of China’s food supply has remarkably changed in the past 53 years. The proportion of basic food such as traditional grain and oil decreased, while high-nutrition food such as meat, poultry and aquatic products gradually increased.

### 3.2. Nutritional Supply and Demand Ratio of Chinese Residents

**Figure 2:**
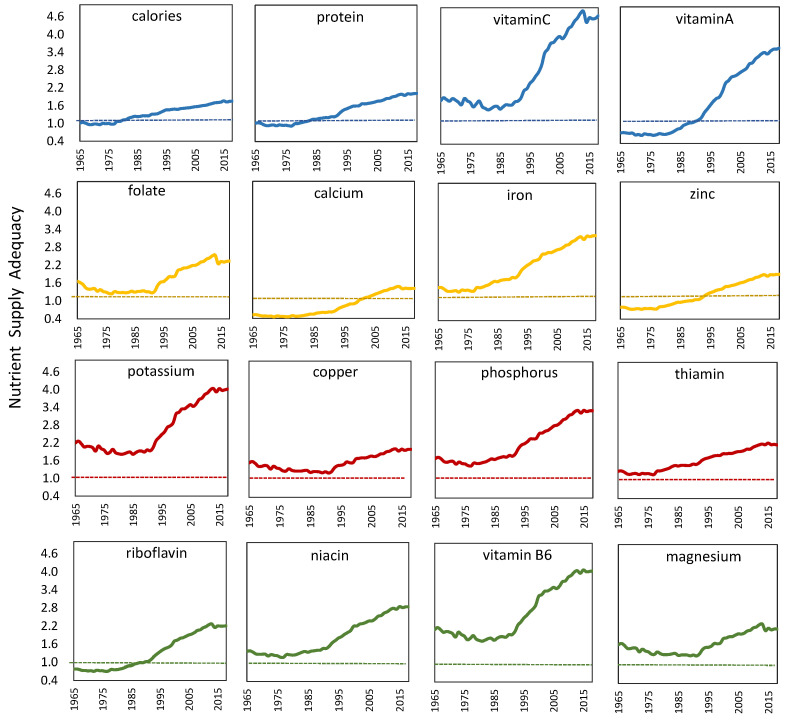
The supply adequacy of different nutrients for Chinese residents from 1965 to 2018.

Figure 2 shows the trend of various nutrients supply over time (1965 to 2018). Overall, the supply adequacy of all nutrients presented an upward trend, but the growth varied fast. It is worth noting that the supply adequacy of vitamin C, folate, iron, potassium, copper, phosphorus, thiamin, niacin, vitamin B6 and magnesium were all higher than one during the whole research period, even though they experienced a slight decline and began to rise in the early 21st century. The supply adequacy of vitamin C and vitamin B6 reached 4 in 2004 and 2012, implying the supply of these two nutrients was more than four times the demand, which could better ensure the supply of nutrients to people. The supply adequacy of copper, thiamin and magnesium were always between one and two, with a slight increase. 

Aquatic products, nuts, and dairy products are respectively rich in copper, magnesium, and riboflavin. It is found that Chinese residents have less intake of aquatic products, nuts and dairy products, making the corresponding nutrient supply insufficient.

Moreover, the supply adequacy of calories and protein were around one in the early stage. Nevertheless, after breaking one in 1977, the ratios began to rise slowly, which shows that the intake of calories and protein is mainly balanced initially, but the intake of some groups may be insufficient due to uneven distribution. As the supply adequacy gradually rises, the intake of calories meets the demand of Chinese residents. However, considering unavoidable uneven distribution, the supply of these two nutrients is still insufficient.

In contrast, the supply of vitamin A, calcium, zinc, and riboflavin experienced significant changes from inadequate intake (supply adequacy <1) to close supply and demand coordination. Vitamin A’s supply adequacy dropped to a low of 0.7 in 1980 and then increased significantly, reaching 3.6 in 2018. For calcium, the supply adequacy of merely 0.5 in 1965 indicated that the supply of calcium was only half of the demand value, significantly insufficient. Its supply adequacy did not break through one and reached a relative balance until the year 2000. Its supply adequacy was also only 1.4 in 2018, which was still a relatively poor state. For zinc and riboflavin, their supply was severely insufficient in 1965 and their supply adequacy gradually exceeded one after 1980s. 

### 3.3. Nutritional Structure of Chinese Residents

**Figure 3:**
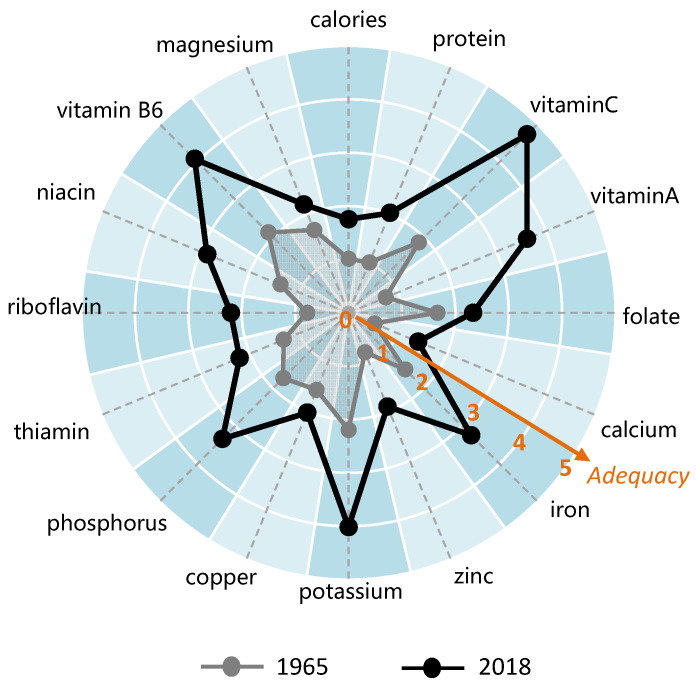
The supply adequacy of different nutrients in food supply of Chinese residents in 1965 and 2018.

Figure 3 shows the supply adequacy of each nutrient by comparing the years 1965 with 2018. Overall, the supply of nutrients increased significantly. Among the sixteen nutrients, twelve had the supply adequacy more than 1, and potassium has the highest supply adequacy with 2.2. On the other hand, the supply adequacy of zinc, riboflavin, vitamin A, and calcium was less than 1, which were 0.8, 0.78, 0.75 and 0.52, respectively. However, the supply adequacy of all nutrients was greater than 1 in 2018. Vitamin C shared the highest supply adequacy with 4.73, and calcium was the lowest with 1.41. The top five nutrients were vitamin C, vitamin B6, potassium, vitamin A and phosphorus. 

In 1965, the supply of vitamin A, calcium, zinc, and riboflavin was incredibly insufficient, while the supply of vitamin C, potassium, and vitamin B6 was more than needed. Compare with 1965, the supply adequacy of all nutrients was increased in 2018. Vitamin C, vitamin A, and vitamin B6 increased the most, their supply adequacy improved 2.87, 2.86, and 1.95, respectively. The supply adequacy of copper, magnesium, and folate have the smallest increase, only 0.46, 0.52, and 0.67, respectively. In general, except for vitamin C, vitamin A, iron, potassium, phosphorus, and vitamin B6, the supply adequacy of other nutrients is relatively low.

## 4. Discussion

### 4.1. Nutrient Profile of the Chinese Food Supply

From 1965 to 2018, the food supply for Chinese residents has shown a steady growth trend. Among them, the meat supply increased the most, while the cereals increased the least. The supply adequacy of most nutrients exceeded one after the 1990s thanks to the rapid development of China’s economy and the great improvement of resident income level during that period. Before the 1990s, the dietary pattern of the Chinese population was grain-dominated. This type of diet was low in diversity, and the supply of some essential nutrients such as protein, calcium, and zinc were extremely low. As the income level began to rise fast in the early 1990s, the supply adequacy of other nutrients except calcium increased greatly in the mid-1990s. In particular, the supply adequacy of vitamin C, vitamin A, potassium, phosphorus, and vitamin B6 showed a sharp increase from 1990.

However, it is worth noting that calcium supply adequacy has been very low throughout the whole study period. Among all the nutrients covered by this study, calcium was the slowest one to reached adequacy greater than one, which was happened in 2000. Even by 2018, the supply adequacy of calcium is still the lowest among all nutrients, only 1.41. Therefore, the food such as high-calcium milk needs to be further increased in China’s food supply. In particular, attention should be paid to the calcium supply of special groups such as children, the elderly, pregnant women, and poor people in less developed regions. In addition, there is a synergistic relationship between nutrients, such as vitamin D and calcium. Vitamin D can promote calcium absorption, while low calcium intake can aggravate vitamin D deficiency [25]. However, due to the limitation of data availability, the supply adequacy of vitamin D was not discussed in this study, and we suggest that future studies could cover more nutrients to provide a more comprehensive analysis of nutritional intake.

Results show that the supply adequacy of vitamin C has soared since the 1990s and reached 4.73 in 2018, which is the highest supply adequacy among all nutrients. However, it should be noted that vitamin C is water-soluble. Compared with Western ways of eating vegetables without cooking, such as salads, Chinese cooking methods such as boiling and stir-frying can significantly lose water-soluble vitamins from vegetables. Although the supply of various vitamins in the Chinese diet is relatively high, this does not mean the intake is adequate. Therefore, besides focusing on a reasonable and healthy dietary pattern, we also need to promote a more scientific and healthy cooking method to minimize the loss of those high-quality nutrients in food during the cooking process.

### 4.2. Policy Implications

The results indicate that the supply of calcium and zinc in current China’s food system especially needs a further enlargement. The National Osteoporosis Foundation (NOF) has assessed the effect of 18 lifestyle factors on peak bone mass. Among them, only dietary calcium intake and dairy products intake were highly rated [26,27]. Currently, the incidence of osteoporotic fractures is still steadily increasing in China [10]. Therefore, it is strongly recommended that Chinese people, especially Chinese adolescents, get adequate calcium intake by promoting dietary intake or taking supplements. At the same time, zinc is widely present in meat, eggs and milk. Therefore, increasing the supply of meat, eggs and milk can effectively mitigate the deficiency in zinc supply. 

Besides changing the food supply structure, fortification and enrichment are also the effective ways to enlarge the nutrient supply. Our results show that folate supply adequacy is still relatively low compared with other nutrients. There are nearly 100 thousand cases of neural tube defects in newborns in China every year [28]. An effective measurement to reduce the risk of neural tube birth defects is to fortify flour with folate. 

On the other hand, nutrient surplus is problematic. The supply of some nutrients is excessive, such as copper, phosphorus, and magnesium. The excess nutrient supply could cause overweight, obesity and other health issues. The report on nutrition and chronic diseases in China [29] claimed that overweight and obesity among Chinese residents are constantly highlighted. The rate of overweight and obesity among residents of all age groups in urban and rural areas continues to rise, with more than half of the adults being overweight or obese. The rates of overweight and obesity among children aged 6–17 even reach 19%.

Besides the actions from governments, raising public awareness of healthy diet is also important. No program can be entirely successful if people do not understand the need for taking action. Therefore, popularizing the nutritional diet knowledge through public education is an effective way to raise people’s awareness of nutrition. We suggest the governments to make more efforts and provide more financial support for the public education of nutritional diet.

### 4.3. Limitations of This Study

This study only considers 16 indicators (total caloric intake and 15 common nutrients) due to data availability. Many other important nutrients such as dietary fibers and unsaturated fatty acid are not covered. We encourage the future studies to build a more detailed database and perform a more comprehensive nutrient assessment. Furthermore, due to data availability, this study performs the assessment from a national perspective and does not consider the heterogeneity of nutrient intake, resulting in that we are unable to identify which population groups are at higher nutrition risks. The investigation focuses on specific population groups is necessary in the further studies to help policymakers to implement targeted supplementation. We strongly suggest the governments and organizations to provide more financial support to those nutrient intake and food consumption investigation projects. Those projects can provide valuable data and information to researchers and policymakers.

## 5. Conclusions

This study reveals the nutrient supply adequacy in China’s food supply in the past half a century based on the nutrient supply and demand. The results show that since 1965, with the rapid development of the social economy and the improvement of people’s living standards, the food supply has shown a steady growth. Meats consumption was growing rapidly while cereals consumption was slowly. From a nutrient perspective, except for calcium, the supply adequacy of remaining nutrients began to rise rapidly in the early 1990s, and all had exceeded one before 1995. The supply adequacies of vitamin C, vitamin A, potassium, phosphorus, and vitamin B6 have experienced dramatic increases since 1990s. Calcium is the severest deficient nutrient among all the studied ones. Its supply adequacy did not exceed 1 until 2000s and was only 1.4 even in 2018, not to mention the unequal occupying of foods and the nutrient loss during cooking. Currently, China is facing double problems of insufficient and excessive intake of nutrients. For nutrients with insufficient intakes, such as calcium and zinc, their supply should be further enlarged through nutritional fortification or targeted supplement. In contrast, for nutrients with excessive intakes, such as iron and magnesium, their daily intake should be controlled to avoid from health issues. Our study reveals the potential risks in China’s food system from a perspective of nutrient supply adequacy. The results are informative for policymakers to implement appropriate interventions to improve China’s public nutrition status.

## Figures and Tables

**Table 1:** The explanation of supply adequacy.

Ratio Value	Explanation
$r_{n}<1$	The supply of this nutrient is seriously inadequate
$r_{n}=1$	The supply of this nutrient is just meeting the demand; considering unequal distribution, this is still an insufficient supply
$r_{n}>1$	The supply of this nutrient exceeds the demand, resulting in a consumer surplus; a greater consumer surplus induces consumer well-being
